# Supplementary material for: Clinical Characteristics and Outcomes of Patients with Primary and Secondary Myelofibrosis According to the Genomic Classification Using Targeted Next-Generation Sequencing
Source: Cancers (Basel). 2023 Jul 31;15(15):3904. doi: 10.3390/cancers15153904 (PMC10417554; doi:10.3390/cancers15153904)
Supplement: Supplementary file 1 [file cancers-15-03904-s001.zip › cancers-2478235-supplementary.pdf]

Supplemental Table S1: Risk factors and stratification of myelofibrosis according to IPSS and MySEC-PM

Risk factors and stratification of myelofibrosis according to IPSS and MySEC

	Risk factors	Risk stratification
**MF-IPSS***	Age >65 years<br>Hemoglobin <10 g/dL<br>Leukocytes >25x10^9/L<br>Circulating blasts ≥1%<br>Constitutional symptoms	Low: 0 factors<br>Intermediate-1: 1 factor<br>Intermediate-2: 2 factors<br>High: 3 or more factors
**MySEC-PM¶**	Age (as a continuum variable)<br>Hemoglobin <11 g/dL (2 points)<br>Platelet count <150x10^9/L (1 point)<br>Circulating blasts ≥3% (2 points)<br>Absence of *CALR* mutation (2 points)<br>Constitutional symptoms (1 point)	Risk stratification (low, intermediate-1, intermediate-2, high) as a sum of the specified points for each variable, except age that is taken into account as a continuum variable

*MF-IPSS: Myelofibrosis International Prognostic Scoring System. Cervantes F, Dupriez B, Pereira A et al. New prognostic scoring system for primary myelofibrosis based on a study of the International Working Group for Myelofibrosis Research and Treatment. Blood. 2009 Mar 26;113(13):2895-901.
¶MySEC-PM: Myelofibrosis Secondary to Polycythemia Vera and Essential Trombocythemia-Prognostic Model. Passamonti F, Giorgino T, Mora B et al. A clinical-molecular prognostic model to predict survival in patients with post polycythemia vera and post essential thrombocythemia myelofibrosis. Leukemia. 2017 Dec;31(12):2726-2731.

Supplemental Table S2: List of genes included in the Next-Generation Sequencing panels that were used

Myeloid Solution by Sophia Genetics (customized)	Oncomine Myeloid Research Assay by Thermo Fisher Scientific
32 genes	40 genes
ABL1 (exons 4-9)	*ABL1* (hotspot regions)
ASXL1 (exons 9, 11, and 12)	***ASXL1*** *(*complete)
BRAF (exon 15)	***BCOR*** *(*complete)
CALR (exon 9)	*BRAF* (hotspot regions)
CBL (exons 8 and 9)	*CALR (*complete)
CEBPA (complete)	***CBL*** (hotspot regions)
CSF3R (complete)	*CEBPA (*complete)
CSNK1A1 (exons 3 and 4)	*CSF3R* (hotspot regions)
DNMT3A (complete)	*DNMT3A* (hotspot regions)
ETV6 (complete)	*ETV6 (*complete)
EZH2 (complete)	***EZH2*** *(*complete)
FLT3 (exons 13, 15 and 20)	*FLT3* (hotspot regions)
HRAS (exons 2 and 3)	***GATA2*** (hotspot regions)
IDH1 (exon 4)	*HRAS* (hotspot regions)
IDH2 (exon 4)	***IDH1*** (hotspot regions)
JAK2 (complete)	***IDH2*** (hotspot regions)
KIT (exons 2, 8-11, 13, 17 and 18)	***IKZF1*** *(*complete)
KMT2A (exons 1-9 and 27)	*JAK2* (hotspot regions)
KRAS (exons 2 and 3)	*KIT* (hotspot regions)
MPL (complete)	***KRAS*** (hotspot regions)
NPM1 (exons 10 and 11)	*MPL* (hotspot regions)
NRAS (exons 2 and 3)	***MYD88*** (hotspot regions)
PTPN11 (exons 3, 7-13)	***NF1*** *(*complete)
RUNX1 (complete)	*NPM1* (hotspot regions)
SETBP1 (exon 4)	***NRAS*** (hotspot regions)
SF3B1 (exons 14-16)	***PHF6*** *(*complete)
SRSF2 (exon 1)	***PRPF8*** *(*complete)
TET2 (complete)	*PTPN11* (hotspot regions)
TP53 (complete)	***RB1*** *(*complete)
U2AF1 (exons 2 and 6)	***RUNX1*** *(*complete)
WT1 (exons 6 and 10)	*SETBP1* (hotspot regions)
ZRSR2 (complete)	*SF3B1* (hotspot regions)
	SH2B3 *(*complete)
	SRSF2 (hotspot regions)
	STAG2 *(*complete)
	*TET2 (*complete)
	*TP53 (*complete)
	U2AF1 (hotspot regions)
	WT1 (hotspot regions)
	ZRSR2 *(*complete)

Analyzed genes that are different in each panel can be found in bold.
Chromatin/splicing genes that are diagnostic of chromatin/splicing mutation category can be found in green. Genes that are not covered by the panels we used but are part of the genomic classification by Grinfeld et al are: *PHF6* (only missing in the 32-gene panel), *CUX1* (missing in

both panels), *GNAS* (missing in both panels), *STAG2* (missing in the 32-gene panel), *BCOR* (missing in the 32-gene panel).

Supplemental Figure S1: Distribution of patients in each molecular category according to risk stratification

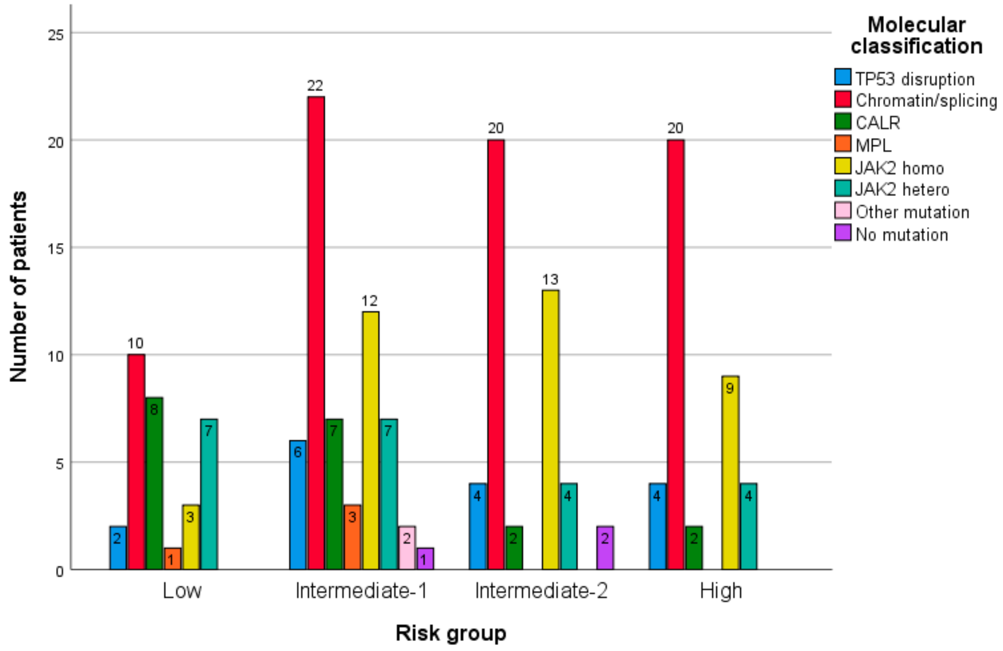

Risk stratification was performed by using IPSS or MySEC for primary and secondary myelofibrosis, respectively. Genomic classification was performed as described by Grinfeld. MF wit *CALR* mutation, MF with *MPL* mutation, MF with heterozygous *JAK2* mutation, and MF with other mutation were classified predominantly in lower risk groups (p not significant for the overall comparison and p=0.002 when high risk genomic groups were pooled together). High risk genomic group included MF with *TP53* disruption/aneuploidy, MF with chromatin/spliceosome mutations, and MF with *JAK2* homozygous mutation.

Supplemental Table S3: Pathogenic and likely pathogenic variants found in the analysis, including allele frequency, manual categorization of the variant and the panel used

The following table shows variants found with NGS analysis that, after manual revision, were considered pathogenic or likely pathogenic and, therefore, taken into account for case classification. Allele frequency is noted in all cases.

Coverage was reviewed for every variant found but not collected systematically during variant notation. However, coverage requirement for considering the variant valid were:

- At least 100 reads in the position where the variant was located, with equilibrated reads in both directions of sequencing (a similar number of reads in both directions). Pathogenic and likely pathogenic variants needed to have an allele frequency of minimum 1% and 30 reads, with equilibrated reads in both directions of sequencing.
- Our quality standards are at least a mean coverage of x500, and ideally over x1000. With a >95% uniformity and on-target sequencing >95%.

Gene	**cDNA**	**Protein**	**VAF**	**Pathogenicity**	**Panel used**	**Coverage**
ASXL1	c.1851_1854dup	p.(Ala619*)	44.6	Pathogenic	Myeloid Solution	
ASXL1	c.2076_2079del	p.(Arg693Hisfs*9)	15.5	Likely pathogenic	Myeloid Solution	
ASXL1	c.1281dupA	p.(Gln428Thrfs*10)	41.9	Likely pathogenic	Myeloid Solution	
ASXL1	c.1549C>T	p.(Gln517*)	13.6	Pathogenic	Myeloid Solution	
ASXL1	c.2278C>T	p.(Gln760*)	41.4	Pathogenic	Myeloid Solution	
ASXL1	c.1900_1922del	p.(Glu635Arsfs*15)	4.0	Pathogenic	Myeloid Solution	
ASXL1	c.1934dupG	p.(Gly646Trpfs*12)	39.3	Pathogenic	Myeloid Solution	
ASXL1	c.1934dup	p.(Gly646Trpfs*12)	40.0	Pathogenic	Myeloid Solution	
ASXL1	c.2292_2273insA	p.(Leu765ThrfsTer9)	32.2	Pathogenic	Myeloid Solution	
ASXL1	c.2597T>G	p.(Leu866*)	13.1	Likely pathogenic	Myeloid Solution	
ASXL1	c.1772dupA	p.(Tyr591*)	3.8	Pathogenic	Myeloid Solution	
ASXL1	c.1773_1776del	p.(Tyr591*)	43.6	Likely pathogenic	Myeloid Solution	
ASXL1	c.3217C>T	p.Arg1073Cys	50.08	Likely Pathogenic	Oncomine	
ASXL1	c.2077C>T	p.Arg693Ter	27.74	Pathogenic	Oncomine	1861
ASXL1	c.2077C>T	p.Arg693Ter	9.75	Likely Pathogenic	Oncomine	
ASXL1	c.2278C>T	p.Gln760Ter	41.43	Pathogenic	Oncomine	1233

ASXL1	c.2332C>T	p.Gln778Ter	53.33	Likely Pathogenic	Oncomine	
ASXL1	c.1902_1924delAGAGGCGGCCACCACTGCCATCG	p.Glu635ArgfsTer15	15.58	Pathogenic	Oncomine	
ASXL1	c.1900_1922delAGAGAGGCGGCCACCACTGCCAT	p.Glu635ArgfsTer15	28.40	Pathogenic	Oncomine	720.6
ASXL1	c.1758_1759insA	p.Gly587ArgfsTer32	16.92	Pathogenic	Oncomine	791.5
ASXL1	c.1889_1910delACCACTGCCATAGAGAGGCGGC	p.His630ProfsTer66	22.29	Pathogenic	Oncomine	720.6
ASXL1	c.2161delC	p.Leu721fs	20.40	Pathogenic	Oncomine	
ASXL1	c.2292_2293insA	p.Leu765ThrfsTer9	32.18	Pathogenic	Oncomine	
ASXL1	c.2066C>G	p.Ser689Ter	4.94	Pathogenic	Oncomine	
ASXL1	c.2693G>A	p.Trp898Ter	14.79	Pathogenic	Oncomine	
CALR	c.1091_1142del	p.(Gly364Glyfs*?)	47.0	Pathogenic	Myeloid Solution	
CALR	c.1099_1150del	p.(Leu367Thrfs*?)	41.0	Pathogenic	Myeloid Solution	
CALR	c.1099_1150del	p.(Leu367Thrfs*?)	43.0	Pathogenic	Myeloid Solution	
CALR	c.1099_1150del	p.(Leu367Thrfs*?)	44.0	Pathogenic	Myeloid Solution	
CALR	c.1099_1150del	p.(Leu367Thrfs*?)	45.0	Pathogenic	Myeloid Solution	
CALR	c.1103_1148del	p.(Lys368Argfs*?)	36.0	Pathogenic	Myeloid Solution	
CALR	c.1154_1155insTTGTC	p.(Lys385Asnfs*?)	31.7	Pathogenic	Myeloid Solution	
CALR	c.1154_1155insTTGTC	p.(Lys385Asnfs*?)	49.8	Pathogenic	Myeloid Solution	
CALR	c.1117_1118delGAinsTT	p.Asp373Phe	44.45	Pathogenic	Oncomine	
CALR	c.1139_1140delAGinsTT	p.Glu380Val	44.54	Pathogenic	Oncomine	
CALR	c.1154_1155insATGTC	p.Glu386fs	78.74	Pathogenic	Oncomine	
CALR	c.1099_1150delCTTAAGGAGGAGGAAGAAGACAAGAAACGCAAAGAGGAGGA	p.Leu367fs	27.57	Pathogenic	Oncomine	

	GGAGGCAGAGG					
CALR	c.1099_1150delCTTAAGGAGGAGGAAGAAGACAAGAAACGCAAAGAGGAGGAGGAGGCAGAGG	p.Leu367fs	29.88	Pathogenic	Oncomine	1087
CALR	c.1099_1150delCTTAAGGAGGAGGAAGAAGACAAGAAACGCAAAGAGGAGGAGGAGGCAGAGG	p.Leu367fs	46.74	Pathogenic	Oncomine	
CALR	c.1099_1150delCTTAAGGAGGAGGAAGAAGACAAGAAACGCAAAGAGGAGGAGGAGGCAGAGG	p.Leu367ThrfsTer46	53.00	Pathogenic	Oncomine	
CALR	c.1099_1150delCTTAAGGAGGAGGAAGAAGACAAGAAACGCAAAGAGGAGGAGGAGGCAGAGG	p.Leu367ThrfsTer46	42.79	Pathogenic	Oncomine	
CALR	c.1099_1150delCTTAAGGAGGAGGAAGAAGACAAGAAACGCAAAGAGGAGGAGGAGGCAGAGG	p.Leu367ThrfsTer46	44.64	Pathogenic	Oncomine	
CALR	c.1099_1150delCTTAAGGAGGAGGAAGAAGACAAGAAACGCAAAGAGGAGGAGGAGGCAGAGG	p.Leu367ThrfsTer46	46.31	Pathogenic	Oncomine	
CALR	c.1099_1150delCTTAAGGAGGAGGAAGAAGACAAGAAACGCAAAGAGGAGGAGGAGGCAGAGG	p.Leu367ThrfsTer46	47.13	Pathogenic	Oncomine	
CALR	c.1099_1150delCTTAAGGAGGAGGAAGAAGACAAGAAACGCAAAGAGGAGGAGGAGGCAGAGG	p.Leu367ThrfsTer46	47.82	Pathogenic	Oncomine	

CALR	c.1099_1150delCTTAAGGAGGAGGAAGAAGACAAGAAACGCAAAGAGGAGGAGGAGGCAGAGG	p.Leu367ThrfsTer46	48.22	Pathogenic	Oncomine	
CALR	c.1099_1150delCTTAAGGAGGAGGAAGAAGACAAGAAACGCAAAGAGGAGGAGGAGGCAGAGG	p.Leu367ThrfsTer46	53	Pathogenic	Oncomine	
CALR	c.1154_1155insTTGTC	p.Lys385AsnfsTer47	38.44	Pathogenic	Oncomine	
CALR	c.1154_1155insTTGTC	p.Lys385AsnfsTer47	38.55	Pathogenic	Oncomine	720.6
CALR	c.1154_1155insTTGTC	p.Lys385AsnfsTer47	62.24	Pathogenic	Oncomine	
CALR	c.1154_1155insTTGTC	p.Lys385AsnfsTer47	80.36	Pathogenic	Oncomine	1861
CALR	c.1154_1155insTTGTC	p.Lys385fs	11.76	Pathogenic	Oncomine	
CALR	c.1154_1155insTTGTC	p.Lys385fs	5.80	Pathogenic	Oncomine	
CBL	c.1259G>A	p.(Arg420Gln)	9.2	Pathogenic	Myeloid Solution	
CBL	C.1111T>G	P.(Tyr371Asp)	21.1	Likely pathogenic	Myeloid Solution	
CBL	c.1211G>T	p.Cys404Phe	13.87	Likely pathogenic	Oncomine	
CBL	c.1111T>G	p.Tyr371Asp	21.09	Likely Pathogenic	Oncomine	893.1
CBL	c.1111T>C	p.Tyr371His	51.41	Pathogenic	Oncomine	
DNMT3A	c.1364_1370dup	p.(Arg458Lysfs*17)	39.7	Likely pathogenic	Myeloid Solution	
DNMT3A	c.2645G>C	p.(Arg882Pro)	41.9	Likely pathogenic	Myeloid Solution	
DNMT3A	c.1792C>T	p.Arg598Ter	7.64	Likely pathogenic	Oncomine	721.2
DNMT3A	c.2207G>A	p.Arg736His	4.20	Likely pathogenic	Oncomine	721.2
DNMT3A	c.2644C>T	p.Arg882Cys	43.46	Pathogenic	Oncomine	
DNMT3A	c.2389A>T	p.Asn797Tyr	6.84	Likely pathogenic	Oncomine	632.4
DNMT3A	c.1718_1718delAinsCAACTG	p.Gln573ProfsTer80	10.01	Likely pathogenic	Oncomine	

DNMT3A	c.2030A>T	p.His677Leu	47.14	Likely pathogenic	Oncomine	
DNMT3A	c.1640T>C	p.Leu547Pro	1.60	Likely pathogenic	Oncomine	773.5
EZH2	c.836_837del	p.(His279Leufs *8)	16.3	Likely pathogenic	Myeloid Solution	
EZH2	c.625+1G>T	p.?	23.93	Pathogenic	Oncomine	
EZH2	c.625+1G>T	p.?	45.93	Pathogenic	Oncomine	
EZH2	c.763G>A	p.Ala255Thr	38.12	Likely pathogenic	Oncomine	
EZH2	c.2051G>A	p.Arg684His	31.27	Likely pathogenic	Oncomine	691.4
EZH2	c.2051G>T	p.Arg684Leu	93.56	Likely pathogenic	Oncomine	
EZH2	c.1733delG	p.Cys578SerfsTer97	19.89	Likely pathogenic	Oncomine	1233
EZH2	c.475G>A	p.Gly159Arg	20.70	Pathogenic	Oncomine	720.6
EZH2	c.446T>G	p.Leu149Arg	6.11	Likely pathogenic	Oncomine	
EZH2	c.833T>G	p.Leu278Ter	27.17	Pathogenic	Oncomine	
EZH2	c.1312_1316delAGTGG	p.Ser438CysfsTer2	2.09	Likely pathogenic	Oncomine	
EZH2	c.2084C>T	p.Ser695Leu	4.63	Likely pathogenic	Oncomine	
IDH1	c.395G>A	p.(Arg132His)	50.0	Pathogenic	Myeloid Solution	
IDH2	c.419G>A	p.(Arg140Gln)	3.2	Pathogenic	Myeloid Solution	
IDH2	c.419G>A	p.(Arg140Gln)	46.7	Pathogenic	Myeloid Solution	
IDH2	c.419G>A	p.Arg140Gln	36.55	Pathogenic	Oncomine	
JAK2	c.1849G>T	p.(Val617Phe)	3.0	Pathogenic	Myeloid Solution	
JAK2	c.1849G>T	p.(Val617Phe)	6.2	Pathogenic	Myeloid Solution	
JAK2	c.1849G>T	p.(Val617Phe)	9.1	Pathogenic	Myeloid Solution	
JAK2	c.1849G>T	p.(Val617Phe)	11	Pathogenic	Myeloid Solution	
JAK2	c.1849G>T	p.(Val617Phe)	15.4	Pathogenic	Myeloid Solution	
JAK2	c.1849G>T	p.(Val617Phe)	16.4	Pathogenic	Myeloid Solution	
JAK2	c.1849G>T	p.(Val617Phe)	20.9	Pathogenic	Myeloid Solution	

JAK2	c.1849G>T	p.(Val617Phe)	26.0	Pathogenic	Oncomine	
JAK2	c.1849G>T	p.(Val617Phe)	26.4	Pathogenic	Myeloid Solution	
JAK2	c.1849G>T	p.(Val617Phe)	30.1	Pathogenic	Myeloid Solution	
JAK2	c.1849G>T	p.(Val617Phe)	30.9	Pathogenic	Myeloid Solution	
JAK2	c.1849G>T	p.(Val617Phe)	31.0	Pathogenic	Myeloid Solution	
JAK2	c.1849G>T	p.(Val617Phe)	36.4	Pathogenic	Myeloid Solution	
JAK2	c.1849G>T	p.(Val617Phe)	39.8	Pathogenic	Myeloid Solution	
JAK2	c.1849G>T	p.(Val617Phe)	40.6	Pathogenic	Myeloid Solution	
JAK2	c.1849G>T	p.(Val617Phe)	41.1	Pathogenic	Oncomine	
JAK2	c.1849G>T	p.(Val617Phe)	42.3	Pathogenic	Myeloid Solution	
JAK2	c.1849G>T	p.(Val617Phe)	42.6	Pathogenic	Oncomine	
JAK2	c.1849G>T	p.(Val617Phe)	43.0	Pathogenic	Myeloid Solution	
JAK2	c.1849G>T	p.(Val617Phe)	44.2	Pathogenic	Myeloid Solution	
JAK2	c.1849G>T	p.(Val617Phe)	45.2	Pathogenic	Myeloid Solution	
JAK2	c.1849G>T	p.(Val617Phe)	46.5	Pathogenic	Myeloid Solution	
JAK2	c.1849G>T	p.(Val617Phe)	47.3	Pathogenic	Myeloid Solution	
JAK2	c.1849G>T	p.(Val617Phe)	48.1	Pathogenic	Myeloid Solution	
JAK2	c.1849G>T	p.(Val617Phe)	48.4	Pathogenic	Myeloid Solution	
JAK2	c.1849G>T	p.(Val617Phe)	53.6	Pathogenic	Myeloid Solution	
JAK2	c.1849G>T	p.(Val617Phe)	54.2	Pathogenic	Myeloid Solution	
JAK2	c.1849G>T	p.(Val617Phe)	55.9	Pathogenic	Myeloid Solution	
JAK2	c.1849G>T	p.(Val617Phe)	56.6	Pathogenic	Oncomine	
JAK2	c.1849G>T	p.(Val617Phe)	57.8	Pathogenic	Myeloid Solution	
JAK2	c.1849G>T	p.(Val617Phe)	67.3	Pathogenic	Myeloid Solution	
JAK2	c.1849G>T	p.(Val617Phe)	68.2	Pathogenic	Myeloid Solution	

JAK2	c.1849G>T	p.(Val617Phe)	71.5	Pathogenic	Myeloid Solution	
JAK2	c.1849G>T	p.(Val617Phe)	77.1	Pathogenic	Oncomine	
JAK2	c.1849G>T	p.(Val617Phe)	81.4	Pathogenic	Myeloid Solution	
JAK2	c.1849G>T	p.(Val617Phe)	84.0	Pathogenic	Myeloid Solution	
JAK2	c.1849G>T	p.(Val617Phe)	88.9	Pathogenic	Myeloid Solution	
JAK2	c.1849G>T	p.(Val617Phe)	89.3	Pathogenic	Myeloid Solution	
JAK2	c.1849G>T	p.(Val617Phe)	89.7	Pathogenic	Myeloid Solution	
JAK2	c.1849G>T	p.(Val617Phe)	92.1	Pathogenic	Myeloid Solution	
JAK2	c.1849G>T	p.(Val617Phe)	92.4	Pathogenic	Myeloid Solution	
JAK2	c.1849G>T	p.(Val617Phe)	92.6	Pathogenic	Myeloid Solution	
JAK2	c.1849G>T	p.(Val617Phe)	96.2	Pathogenic	Myeloid Solution	
JAK2	c.1849G>T	p.(Val617Phe)	97.9	Pathogenic	Myeloid Solution	
JAK2	c.1849G>T	p.(Val617Phe)	97.9	Pathogenic	Myeloid Solution	
JAK2	c.1849G>T	p.(Val617Phe)	99.7	Pathogenic	Myeloid Solution	
JAK2	c.1849G>T	p.Val617Phe	17.9	Pathogenic	Oncomine	
JAK2	c.1849G>T	p.Val617Phe	55.8	Pathogenic	Oncomine	
JAK2	c.1849G>T	p.Val617Phe	72.7	Pathogenic	Oncomine	
JAK2	c.1849G>T	p.Val617Phe	10.41	Pathogenic	Oncomine	1113
JAK2	c.1849G>T	p.Val617Phe	16.08	Pathogenic	Oncomine	903.2
JAK2	c.1849G>T	p.Val617Phe	2.83	Pathogenic	Oncomine	
JAK2	c.1849G>T	p.Val617Phe	20.91	Pathogenic	Oncomine	
JAK2	c.1849G>T	p.Val617Phe	27.82	Pathogenic	Oncomine	
JAK2	c.1849G>T	p.Val617Phe	29.91	Pathogenic	Oncomine	
JAK2	c.1849G>T	p.Val617Phe	3.82	Pathogenic	Oncomine	834.8
JAK2	c.1849G>T	p.Val617Phe	3.88	Pathogenic	Oncomine	721.2
JAK2	c.1849G>T	p.Val617Phe	32.78	Pathogenic	Oncomine	701.1
JAK2	c.1849G>T	p.Val617Phe	34.45	Pathogenic	Oncomine	
JAK2	c.1849G>T	p.Val617Phe	38.61	Pathogenic	Oncomine	
JAK2	c.1849G>T	p.Val617Phe	39.37	Pathogenic	Oncomine	1206

JAK2	c.1849G>T	p.Val617Phe	39.82	Pathogenic	Oncomine	
JAK2	c.1849G>T	p.Val617Phe	4.10	Pathogenic	Oncomine	632.4
JAK2	c.1849G>T	p.Val617Phe	4.40	Pathogenic	Oncomine	770.5
JAK2	c.1849G>T	p.Val617Phe	40.59	Pathogenic	Oncomine	
JAK2	c.1849G>T	p.Val617Phe	41.07	Pathogenic	Oncomine	
JAK2	c.1849G>T	p.Val617Phe	42.50	Pathogenic	Oncomine	
JAK2	c.1849G>T	p.Val617Phe	43.55	Pathogenic	Oncomine	
JAK2	c.1849G>T	p.Val617Phe	44.18	Pathogenic	Oncomine	917.8
JAK2	c.1849G>T	p.Val617Phe	45.05	Pathogenic	Oncomine	
JAK2	c.1849G>T	p.Val617Phe	46.09	Pathogenic	Oncomine	963.4
JAK2	c.1849G>T	p.Val617Phe	46.50	Pathogenic	Oncomine	
JAK2	c.1849G>T	p.Val617Phe	47.31	Pathogenic	Oncomine	
JAK2	c.1849G>T	p.Val617Phe	5.31	Pathogenic	Oncomine	780.4
JAK2	c.1849G>T	p.Val617Phe	52.50	Pathogenic	Oncomine	
JAK2	c.1849G>T	p.Val617Phe	52.92	Pathogenic	Oncomine	1031
JAK2	c.1849G>T	p.Val617Phe	6.32	Pathogenic	Oncomine	773.5
JAK2	c.1849G>T	p.Val617Phe	62.60	Pathogenic	Oncomine	
JAK2	c.1849G>T	p.Val617Phe	64.11	Pathogenic	Oncomine	1048
JAK2	c.1849G>T	p.Val617Phe	65.06	Pathogenic	Oncomine	
JAK2	c.1849G>T	p.Val617Phe	66.72	Pathogenic	Oncomine	1043
JAK2	c.1849G>T	p.Val617Phe	77.88	Pathogenic	Oncomine	1048
JAK2	c.1849G>T	p.Val617Phe	78.37	Pathogenic	Oncomine	
JAK2	c.1849G>T	p.Val617Phe	78.82	Pathogenic	Oncomine	
JAK2	c.1849G>T	p.Val617Phe	80.30	Pathogenic	Oncomine	1084
JAK2	c.1849G>T	p.Val617Phe	81.41	Pathogenic	Oncomine	
JAK2	c.1849G>T	p.Val617Phe	87.21	Pathogenic	Oncomine	950
JAK2	c.1849G>T	p.Val617Phe	87.25	Pathogenic	Oncomine	876.6
JAK2	c.1849G>T	p.Val617Phe	90.12	Pathogenic	Oncomine	
JAK2	c.1849G>T	p.Val617Phe	92.15	Pathogenic	Oncomine	
JAK2	c.1849G>T	p.Val617Phe	92.36	Pathogenic	Oncomine	
JAK2	c.1849G>T	p.Val617Phe	94.46	Pathogenic	Oncomine	
JAK2	c.1849G>T	p.Val617Phe	94.59	Pathogenic	Oncomine	
JAK2	c.1849G>T	p.Val617Phe	97.89	Pathogenic	Oncomine	
KRAS	c.437C>T	p.(Ala146Val)	34.4	Pathogenic	Myeloid Solution	
KRAS	c.176C>G	p.(Ala59Gly)	12.1	Likely pathogenic	Myeloid Solution	

KRAS	c.57G>C	p.(Leu19Phe)	5.2	Likely pathogenic	Myeloid Solution	
KRAS	c.70A>C	p.Ile24Leu	5.21	Pathogenic	Oncomine	1233
KRAS	c.57G>C	p.Leu19Phe	5.20	Pathogenic	Oncomine	1233
MPL	c.1544G>T	p.Trp515Leu	50.24	Pathogenic	Oncomine	
MPL	c.1544G>T	p.Trp515Leu	66.44	Pathogenic	Oncomine	
MPL	c.1543_1544delTGinsAA	p.Trp515Lys	54.38	Pathogenic	Oncomine	893.1
MPL	c.1895G>C	p.Trp632Ser	23.09	Pathogenic	Oncomine	
MPL	c.1771T>G	p.Tyr591Asp	1.63	Likely pathogenic	Oncomine	1031
MPL	c.1771T>G	p.Tyr591Asp	46.66	Likely pathogenic	Oncomine	701.1
MPL	c.1771T>G	p.Tyr591Asp	58.45	Likely pathogenic	Oncomine	
NRAS	c.175G>A	p.Ala59Thr	9.68	Pathogenic	Oncomine	1441
PTPN11	c.1471C>T	p.Pro491Ser	44.92	Likely pathogenic	Oncomine	
PTPN11	c.1504T>C	p.Ser502Pro	29.39	Pathogenic	Oncomine	
RUNX1	c.511G>A	p.(Asp171Asn)	3.7	Likely pathogenic	Myeloid Solution	
RUNX1	c.1308del	p.(Thr437Profs*?)	4.0	Likely pathogenic	Myeloid Solution	
RUNX1	c.508+2T>C	p.?	5.16	Likely pathogenic	Oncomine	950
SETBP1	c.2602G>A	p.(Asp868Asn)	13.1	Pathogenic	Myeloid Solution	
SETBP1	c.2602G>A	p.Asp868Asn	26.95	Pathogenic	Oncomine	
SETBP1	c.2602G>A	p.Asp868Asn	49.89	Pathogenic	Oncomine	
SETBP1	c.2608G>A	p.Gly870Ser	33.90	Likely pathogenic	Oncomine	
SF3B1	c.2098A>G	p.(Lys700Glu)	44.9	Pathogenic	Myeloid Solution	
SF3B1	c.2242A>G	p.(Lys748Glu)	32.7	Likely pathogenic	Myeloid Solution	
SF3B1	c.1877A>G	p.Asn626Ser	2.60	Pathogenic	Oncomine	876.6
SF3B1	c.1998G>C	p.Lys666Asn	21.52	Pathogenic	Oncomine	
SF3B1	c.1998G>T	p.Lys666Asn	33.43	Pathogenic	Oncomine	1206
SF3B1	c.1998G>T	p.Lys666Asn	36.43	Pathogenic	Oncomine	
SF3B1	c.1997A>C	p.Lys666Thr	42.43	Pathogenic	Oncomine	641.2
SRSF2	c.284C>G	p.(Pro95Arg)	32.3	Pathogenic	Myeloid Solution	

SRSF2	c.284C>G	p.(Pro95Arg)	40.9	Pathogenic	Myeloid Solution	
SRSF2	c.284C>G	p.(Pro95Arg)	49.5	Pathogenic	Myeloid Solution	
SRSF2	c.284C>A	p.(Pro95His)	39.3	Likely pathogenic	Myeloid Solution	
SRSF2	c.284C>A	p.(Pro95His)	45.8	Pathogenic	Myeloid Solution	
SRSF2	c.284C>A	p.(Pro95His)	47.2	Pathogenic	Myeloid Solution	
SRSF2	c.284C>T	p.(Pro95Leu)	45.5	Likely pathogenic	Myeloid Solution	
SRSF2	c.284C>T	p.(Pro95Leu)	46.4	Likely pathogenic	Myeloid Solution	
SRSF2	c.102C>A, c.-1294G>T	p.Phe34Leu, p.?	1.66	likely pathogenic	Oncomine	1031
SRSF2	c.283C>G	p.Pro95Ala	2.27	Pathogenic	Oncomine	728.5
SRSF2	c.283C>G	p.Pro95Ala	42.55	Pathogenic	Oncomine	
SRSF2	c.284C>G	p.Pro95Arg	44.19	Likely pathogenic	Oncomine	
SRSF2	c.283C>G	p.Pro95Arg, p.?	44.57	Pathogenic	Oncomine	
SRSF2	c.284C>A	p.Pro95His	46.84	Pathogenic	Oncomine	
SRSF2	c.284C>A	p.Pro95His	50.65	Pathogenic	Oncomine	1113
SRSF2	c.284C>A, c.-1476G>T	p.Pro95His, p.?	48.10	Pathogenic	Oncomine	
SRSF2	c.284C>A, c.-1476G>T	p.Pro95His, p.?	57.25	Pathogenic	Oncomine	
TET2	c.4546C>T	p.(Arg1516*)	47.3	Pathogenic	Myeloid Solution	
TET2	c.5134A>T	p.(Arg1712*)	4.0	Likely pathogenic	Myeloid Solution	
TET2	c.436del	p.(Asp146Ilefs*6)	34.1	Likely pathogenic	Myeloid Solution	
TET2	c.483dup	p.(Asp162Argfs*9)	3.9	Pathogenic	Myeloid Solution	
TET2	c.2200C>T	p.(Gln734*)	45.3	Likely pathogenic	Myeloid Solution	
TET2	c.2662C>T	p.(Gln888*)	6.1	Likely pathogenic	Myeloid Solution	
TET2	c.3718_3719del	p.(Leu1240Glyfs*2)	2.3	Likely pathogenic	Myeloid Solution	
TET2	c.4590_4604del	p.(Leu1531_Pro1535del)	47	Likely pathogenic	Myeloid Solution	
TET2	c.3065delA	p.(Lys1022ArgfsTer11)	49.2	Pathogenic	Myeloid Solution	

TET2	c.875_876insT	p.(Ser293GlufsTer2)	16.4	Likely pathogenic	Myeloid Solution	
TET2	c.94_95insAGCTA	p.(Thr32LysTer19)	41.9	Likely pathogenic	Myeloid Solution	
TET2	c.576C>G	p.(Tyr192*)	82.2	Likely pathogenic	Myeloid Solution	
TET2	c.3501-1G>A	p.?	42.13	Likely pathogenic	Oncomine	
TET2	c.4427delC	p.Ala1476ValfsTer95	34.51	Likely pathogenic	Oncomine	1600
TET2	c.3646C>T	p.Arg1216Ter	49.20	Likely pathogenic	Oncomine	
TET2	c.3782G>A	p.Arg1261His	1.42	Likely pathogenic	Oncomine	
TET2	c.3784C>T	p.Arg1262Trp	6.33	Likely pathogenic	Oncomine	917.8
TET2	c.4062_4063delAG	p.Arg1354SerfsTer46	35.89	Likely pathogenic	Oncomine	
TET2	c.4546C>T	p.Arg1516Ter	28.77	Likely pathogenic	Oncomine	1113
TET2	c.1648C>T	p.Arg550Ter	43.87	Pathogenic	Oncomine	950
TET2	c.2256_2259delTAAA	p.Asn752LysfsTer60	12.94	Pathogenic	Oncomine	1113
TET2	c.3812_3813insG	p.Cys1271fs	39.22	Pathogenic	Oncomine	
TET2	c.4042C>T	p.Gln1348Ter	38.03	Likely pathogenic	Oncomine	876.6
TET2	c.2305C>T	p.Gln769Ter	4.65	Pathogenic	Oncomine	
TET2	c.2428C>T	p.Gln810Ter	49.86	Likely pathogenic	Oncomine	
TET2	c.2671C>T	p.Gln891Ter	72.51	Likely pathogenic	Oncomine	933.4
TET2	c.5263G>T	p.Glu1755Ter	43.92	Likely pathogenic	Oncomine	
TET2	c.550_551delGAinsT	p.Glu184CysfsTer23	58.52	Likely pathogenic	Oncomine	
TET2	c.4544delT	p.Leu1515CysfsTer56	43.83	Pathogenic	Oncomine	
TET2	c.4936_4937insAGTGGACAACTGCTCCCCATATC	p.Leu1646fs	16.91	Likely pathogenic	Oncomine	
TET2	c.3065delA	p.Lys1022ArgfsTer11	49.19	Pathogenic	Oncomine	917.8
TET2	c.976A>T	p.Lys326Ter	1.90	Likely pathogenic	Oncomine	1031

TET2	c.3854_3856delTCT	p.Phe1285del	3.43	Likely pathogenic	Oncomine	
TET2	c.1970_1971delCAinsT	p.Ser657PhefsTer43	13.15	Likely pathogenic	Oncomine	
TET2	c.94_95insAGCTA	p.Thr32LysfsTer19	41.87	Likely pathogenic	Oncomine	
TET2	c.3594delG	p.Val1199TrpfsTer27	8.86	Likely pathogenic	Oncomine	
TET2	c.3637G>A	p.Val1213Met	53.01	Pathogenic	Oncomine	1031
TP53	c.916C>T	p.(Arg306*)	31.0	Pathogenic	Myeloid Solution	
TP53	c.182A>G	p.(Asp61Gly)	9.0	Pathogenic	Myeloid Solution	
TP53	c.824G>A	p.(Cys275Tyr)	45.7	Pathogenic	Myeloid Solution	
TP53	c.707A>G	p.(Tyr236Cys)	14.1	Pathogenic	Myeloid Solution	
TP53	c.376-2A>G	p.?	1.56	Pathogenic	Oncomine	800.6
TP53	c.578A>G	p.His193Arg	50.09	Pathogenic	Oncomine	1113
TP53	c.646G>A	p.Val216Met	7.27	Pathogenic	Oncomine	
TP53	c.559+1G>A		4.0	Pathogenic	Myeloid Solution	
TP53	c.559+2T>C		10.4	Likely pathogenic	Myeloid Solution	
U2AF1	c.470A>G	p.(Gln157Arg)	43.2	Pathogenic	Myeloid Solution	
U2AF1	c.470A>G	p.(Gln157Arg)	49.4	Pathogenic	Myeloid Solution	
U2AF1	c.470A>C	p.(Gln157Pro)	2.1	Pathogenic	Myeloid Solution	
U2AF1	c.470A>C	p.(Gln157Pro)	44.9	Pathogenic	Myeloid Solution	
U2AF1	c.101C>T	p.(Ser34Phe)	43.6	Pathogenic	Myeloid Solution	
U2AF1	c.470A>C, c.251A>C	p.Gln157Pro, p.Gln84Pro	15.41	Pathogenic	Oncomine	
U2AF1	c.470A>C, c.251A>C	p.Gln157Pro, p.Gln84Pro	21.19	Pathogenic	Oncomine	1084
U2AF1	c.470A>C, c.251A>C	p.Gln157Pro, p.Gln84Pro	36.84	Pathogenic	Oncomine	
U2AF1	c.101C>T, c.-8623C>T	p.Ser34Phe, p.?	15.64	Pathogenic	Oncomine	
U2AF1	c.101C>T, c.-8623C>T	p.Ser34Phe, p.?	43.63	Pathogenic	Oncomine	
ZRSR2	c.376C>T	p.(Arg126*)	4.4	Likely pathogenic	Myeloid Solution	

ZRSR2	c.196A>T	p.(Arg66*)	6.5	Likely pathogenic	Myeloid Solution	
ZRSR2	c.202_203dup	p.(Gln69Glyfs*10)	3.2	Likely pathogenic	Myeloid Solution	
ZRSR2	c.330delA	p.Gln110HisfsTer55	29.44	Likely pathogenic	Oncomine	
ZRSR2	c.826T>C	p.Ser276Pro	6.59	Pathogenic	Oncomine	1233

Supplemental Table S4. Multivariate analysis of risk factors for survival according to type of myelofibrosis

Multivariate analysis of risk factors for survival according to type of myelofibrosis								
	PMF N=81		Post-PV MF N=53		Post-ET MF N=41		Total MF N=175	
	HR (95%CI)	p	HR (95%CI)	p	HR (95%CI)	p	HR (95%CI)	p
Molecular high-risk	3.6 (1.4-9.3)	0.008	1.9 (0.8-4.5)	0.1	1.5 (0.2-11.7)	0.7	2.6 (1.4-4.9)	0.003
IPSS	2.1 (1.5-3.0)	<0.001	-	-	-	-	1.8 (1.4-2.4)	<0.001
MYSEC	-	-	1.9 (1.3-2.8)	0.001	2.4 (1.3-4.4)	0.006	-	-

PMF: primary myelofibrosis. Post-PV MF: post-polycythemia vera myelofibrosis. Post-ET MF: post-essential thrombocythemia myelofibrosis. MF: myelofibrosis. HR: hazard ratio. CI: confidence intervals. Molecular high-risk: MF with *TP53* disruption of aneuploidy, MF with spliceosome or chromatin mutations and MF with homozygous *JAK2* mutation. IPSS: international prognosis scoring system. MYSEC: myelofibrosis-secondary-to-PV-and ET-prognostic-model

Supplemental Table S5. Multivariate analysis of risk factors for progression to acute leukemia according to type of myelofibrosis

Multivariate analysis of risk factors for progression to acute leukemia according to type of myelofibrosis								
	PMF N=81		Post-PV MF N=53		Post-ET MF N=41		Total MF N=175	
	HR (95%CI)	p	HR (95%CI)	p	HR (95%CI)	p	HR (95%CI)	p
MF with *TP53* disruption or aneuploidy	4.2 (0.5-36)	0.2	10.1 (2.1-48.4)	0.004	7.8 (1.1-56.6)	0.04	5.7 (1.8—18.4)	0.004
IPSS	1.6 (0.7-3.8)	0.2	-	-	-	-	2.0 (1.1-3.5)	0.01
MYSEC			2.3 (1.1-4.7)	0.02	0.9 (0.3-2.5)	0.9	-	-

PMF: primary myelofibrosis. Post-PV MF: post-polycythemia vera myelofibrosis. Post-ET MF: post-essential thrombocythemia myelofibrosis. MF: myelofibrosis. HR: hazard ratio. CI: confidence intervals. IPSS: international prognosis scoring system. MYSEC: myelofibrosis-secondary-to-PV-and ET-prognostic-model
